# Intermittent fasting reduces glaucomatous damage in an HSP27 autoimmune mouse model

**DOI:** 10.3389/fncel.2025.1690991

**Published:** 2026-01-20

**Authors:** Dominik L. Maler, Sabrina Reinehr, Leonie Deppe, H. Burkhard Dick, Stephanie C. Joachim

**Affiliations:** Experimental Eye Research Institute, University Eye Hospital, Ruhr-University Bochum, Bochum, Germany

**Keywords:** diet, fasting, glaucoma, heat shock proteins, HSP27, macroglia, retinal ganglion cells

## Abstract

**Background:**

Currently, the treatment of glaucoma is limited to reducing intraocular pressure since other involved pathomechanisms are not well understood yet. Evidence points to an immune-mediated component in disease development. For example, elevated antibody levels against heat shock protein 27 (HSP27) were detected in glaucoma patients. In mice, we previously noted glaucoma-like damage after an intravitreal HSP27 injection. Now, we aimed to investigate if intermittent fasting protects from this glaucomatous damage.

**Methods:**

CD1 mice were intravitreally injected with HSP27 into one eye. The contralateral eye served as a control. After injections, half of the animals received food ad libitum (no diet). The other half fasted, hence access to food was denied for 24 h at three days per week (diet). The animals were weighed weekly. Retinal thickness was analyzed via optical coherence tomography (OCT) after 4 weeks (*n* = 7 eyes/group). Via immunohistology, retinal ganglion cells (RGCs), apoptotic cells, macroglia, microglia/macrophages, tumor necrosis factor (TNFα), and interleukin (IL)-1β were analyzed (*n* = 6 eyes/group). Corresponding markers were examined with RT-qPCR (*n* = 4 samples/group). In addition, microarray assays were performed from serum samples from mice with diet or with no diet (*n* = 6 samples/group).

**Results:**

The weight and OCT measurements revealed no differences between the groups. HSP27 retinas had significantly lower RGC numbers as well as decreased *Rbmps* mRNA levels compared to controls, while HSP27+diet retinas displayed similar RGC counts as controls. No difference was observed in apoptotic markers. The macroglia^+^ area was increased in HSP27 tissue, while the HSP27+diet group showed no difference to controls. The number of microglia was not altered after HSP27 injection but was lower in HSP27+diet retinas. *Tnfa* and *Il1b* expression levels were downregulated in HSP27+diet samples compared to control as well as HSP27 tissue. Moreover, different pro-inflammatory cytokines, including IL-1β and IL-6, were lower in the serum of diet mice compared to no diet ones.

**Conclusion:**

Intravitreal injection of HSP27 resulted in RGC loss and was associated with gliosis. In contrast, intermittent fasting conferred neuroprotective effects, likely by modulating neuroinflammatory pathways, and hence protected RGCs from damage. These findings highlight intermittent fasting as a potential adjunctive therapeutic strategy for glaucoma management.

## 1 Introduction

Glaucoma has seen a significant increase in the number of patients in recent years (Zhang et al., 2021). By 2040, over 110 million people worldwide are expected to suffer from this disease. With the rising number of cases, the proportion of individuals who lose their vision is also increasing (Tham et al., 2014). Thus, glaucoma is the second most common cause of blindness and one of the most severe conditions in ophthalmology (Quigley and Broman, 2006). However, current treatment options are limited to lowering intraocular pressure, which is often insufficient.

Evidence points to an immune-mediated component. Studies have shown an increased concentration of antibodies against heat shock protein (HSP) in the serum of glaucoma patients. For example, serum samples from normal as well as high-pressure glaucoma patients displayed elevated autoantibody titers against small HSPs, including *α*- and *β*-crystalline as well as HSP27 (Tezel et al., 1998). In samples from Japan and the USA elevated antibody levels against small HSPs in glaucoma patients were found, with even higher levels noted in those with normal-pressure glaucoma (Wax et al., 2001). HSPs are part of the immune system, they are divided into families alongside their molecular weight and under physiological circumstances they are usually expressed in most cells (Lindquist and Craig, 1988). Furthermore, they are molecular chaperons protecting cells from stress and have an anti-apoptotic activity in neurons. Usually, these chaperons enable protein folding and unfolding, but under stress the concentration of HSPs increase to function as a protection for the cell (Lindquist and Craig, 1988; D'Souza and Brown, 1998). Also, in the central nervous system large quantities of HSPs can be found, especially in oligodendrocytes, astrocytes, and neurons which lead to the conclusion that the protective role of these chaperons is very crucial. The accumulation of HSPs in cells was observed due to degenerative, inflammatory, or toxic-metabolic states (D'Souza and Brown, 1998; Plumier et al., 1997). Extracellularly, increased HSPs levels can lead to the activation of the immune system. Thereby, the immune reaction induces cell death of neurons (De Maio and Vazquez, 2013). In agreement with this, Wax et al. (2008) demonstrated glaucoma-like damage after systemic HSP injection in rats. Preliminary work by our group has already demonstrated that local intravitreal injection of HSP27 leads to retinal ganglion cell (RGC) loss in rats (Grotegut et al., 2020). We then successfully transferred this HSP27 model to mice, where, similarly to rats, degeneration of RGCs and the optic nerve occurs after a single intravitreal injection (Erb et al., 2023).

The question arises whether non-pharmacological approaches could also help to slow the progression of this neurodegenerative disease. Studies have, for instance, investigated a potential link between fasting and neurodegeneration. Kawai et al. (2001) have already illustrated that the loss of RGCs in the periphery of the retina could be suppressed in both young as well as old mice through caloric restriction. Guo et al. (2016) also observed the suppression of neurodegeneration in mice. Furthermore, improvements in visual performance were detected under a fasting regimen, as reflected in a better electrophysiological response in the electroretinogram. This further emphasizes the significance of novel therapeutic approaches. Additionally, increased regenerative capacity of the inner retinal layer was noted in mice after fasting (Kong et al., 2012). A review article by Francisco et al. (2020) compared the significance of various dietary patterns in the four main ophthalmic diseases. They concluded that controlled studies and experimental work are necessary, as associations between diet and neurodegeneration were highlighted in this article. Furthermore, an animal study revealed that a diet could reduce the concentration of reactive oxygen species (ROS) (Cooper et al., 2018). ROS are often associated with mitochondrial dysfunction, which in turn leads to the degeneration of RGCs and axonal degeneration (Munemasa et al., 2010).

In our study, we now aimed to elaborate whether intermittent fasting could preserve the retina from glaucoma-like damage after intravitreal HSP27 in mice. While HSP27 injections caused RGC loss and gliosis, fasting mitigated these effects and normalized inflammatory markers both in the retina and serum.

## 2 Materials and methods

### 2.1 Animals

Female and male CD1 mice (6-week-old; Charles River, Sulzfeld, Germany) were utilized in this study. All mice were kept in an environmentally controlled setting with unrestricted access to water and maintained on a 12-h light–dark cycle.

28 days after injection, mice were sacrificed by carbon dioxide inhalation according to the American Veterinary Medical Association guidelines (Leary et al., 2020) and approved by the Animal Welfare Commission of North Rhine-Westphalia (approval code: 81.02.04.2020.A084). The study was conducted in accordance with the guidelines of the Association for Research in Vision and Ophthalmology for animal experiments. All mice were monitored for signs of distress throughout their life. Animals would have been excluded if they showed severe health impairments unrelated to the experimental procedure or if humane endpoints were reached before study completion. Moreover, intraocular bleeding or severe inflammation of the eyes, including redness, swelling, or viscous eyes, were used as exclusion criteria. No mice met these criteria and no animals died spontaneously or required early euthanasia before the planned endpoint.

### 2.2 HSP27 injection

**Figure 1:**
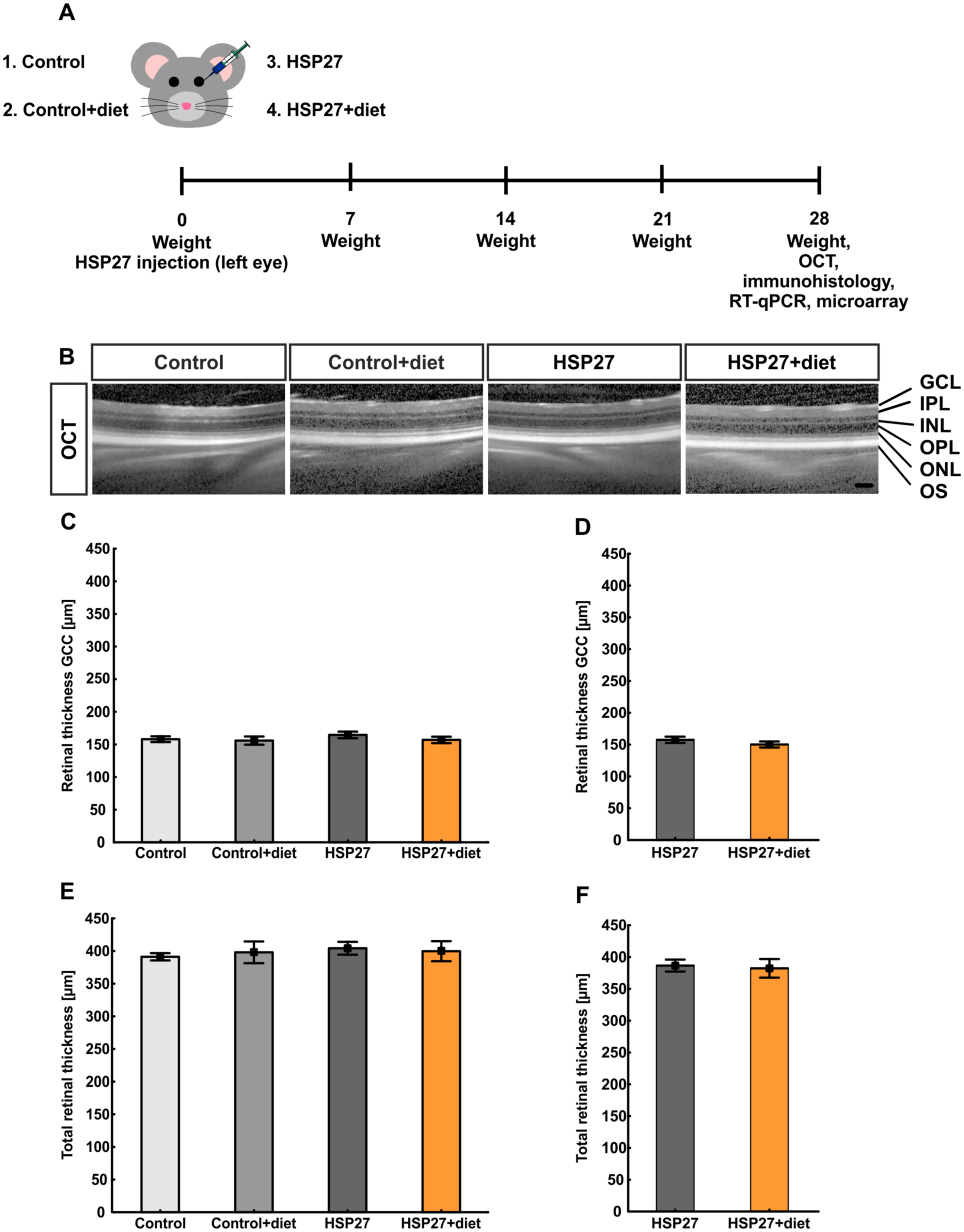
**(A)** Study design including the four groups: control, control+diet, HSP27, and HSP27+diet retinas. For diet animals, food was restricted for 24 h on Mondays, Wednesdays, and Fridays. The animals were weighed weekly. 28 days after HSP27 injection, all eyes and serum samples were processed for subsequent analyses. **(B)** Representative OCT images for each group. **(C)** The thickness of the GCC did not differ between all groups. **(D)** The GCC thickness remained unaltered between HSP27+diet and HSP27 eyes. **(E)** OCT evaluation showed no alteration in total retinal thickness between the groups. **(F)** Also, no difference was noted between HSP27+diet and HSP27 retinas. GCL, ganglion cell layer; IPL, inner plexiform layer; INL, inner nuclear layer; OPL, outer plexiform layer; ONL, outer nuclear layer; OS, outer segment. Values are mean ± SEM, *n* = 7 eyes/group. Scale bar: 200 μm.

Anesthesia for mice was induced in a low-stress manner in an airtight container with 4% isoflurane (CP-Pharma, Burgdorf, Germany) until relevant reflexes have ceased. Anesthesia was maintained using a specially designed mask with a gas mixture of approx. 1.5% isoflurane and oxygen. Furthermore, a topical anesthetic (4 mg/mL, Conjuncain, Bausch & Lomb, Rochester, NY, USA) was applied to the eye, followed by a mydriatic agent (5 mg/mL, Tropicamide, Pharma Stulln, Stulln, Germany) to dilate the pupil. The HSP27 protein (cat. HSP0503; Lot: 097102, AtGen, Yatap-dong, South Korea) was pre-dissolved in 20 mM phosphate-buffered saline (PBS, pH 7.5). Under a stereomicroscope (Zeiss, Oberkochen, Germany), a 32-gauge needle (Hamilton, Reno, NV, USA) was used to inject 1 μL of 0.6 μg/μL HSP27 solution into one eye per animal (Erb et al., 2023). The non-injected contralateral eyes served as controls (Figure 1A). Following the injection, the animals were closely monitored to ensure they remained in good health.

### 2.3 Diet protocol

Directly after HSP27 injection, animals were divided into two groups, namely a control group that had continual access to food (no diet) and a diet group that was deprived of food three times per week. In detail, food was omitted on Mondays, Wednesdays, and Fridays for 24 hours (h; diet). All mice were weighed weekly for the duration of 4 weeks (Figure 1A).

### 2.4 Optical coherence tomography

At the end of the study, mice were anesthetized with ketamine (120 mg/mL, Ratiopharm, Ulm, Germany) and xylazine (16 mg/kg, Bayer Health Care, Leverkusen, Germany). Optical coherence tomography (OCT) images were acquired 4 weeks after the HSP27 injection using the iVivo® LAB OCT (OcuScience, Henderson, NV, USA). For the analysis of retinal thickness, both central and peripheral images were captured. The ganglion cell complex (GCC), including the retinal nerve fiber layer, ganglion cell layer (GCL), and inner plexiform layer (IPL) as well as the total retinal thickness (GCL, IPL, inner nuclear layer (INL), outer plexiform layer (OPL), and outer nuclear layer (ONL)) for all groups (*n* = 7 eyes/group) was measured perpendicularly to the individual layers using ImageJ software (NIH, Bethesda, MD, USA). The means were calculated based on five individual measurements per eye (Berger et al., 2014; Petrikowski et al., 2021).

### 2.5 Immunohistology and evaluation

Immediately after OCT analysis (4 weeks after injection), all mice were euthanized by carbon dioxide inhalation (flow rate 30% of chamber volume/min). Eyes for immunohistology were fixed in 4% paraformaldehyde (Merck, Darmstadt, Germany) for one hour (*n* = 6 eyes/group). The tissue was then cryo-preserved in 30% sucrose overnight before being frozen and embedded in NEG-50 Tissue Tek medium (Thermo Fisher Scientific, Waltham, MA, USA). 10 μm thick cross-sections were cut with a cryostat (Thermo Fisher Scientific) for further staining. Specific immunofluorescence antibodies were employed to identify the different cell types in the retina (Table 1). Retinal sections were first blocked with a solution containing 20% donkey serum and 0.1% Triton-X in PBS. The sections were then incubated overnight at room temperature with specific primary antibodies. The following day, the sections were incubated with the appropriate secondary antibodies for one hour (Table 1). For all staining procedures, 4′,6-diamidino-2-phenylindole (DAPI, Serva Electrophoresis, Heidelberg, Germany) was used to label cell nuclei. Negative controls were performed for each staining by using only secondary antibodies (Grotegut et al., 2021).

**Table 1:** Primary antibodies listed in alphabetical order and corresponding secondary antibodies used for immunohistology.

Primary antibodies					Secondary antibodies				
Antibody	Company	Catalog number	Clonality	Dilution	Antibody	Company	Catalog number	Clonality	Dilution
CD3-FITC (rat)	eBioscience	11–0030	Monoclonal	1:100	Not applicable				
Cleaved caspase 3 (rabbit)	Sigma-Aldrich	C8487	Polyclonal	1:300	Donkey anti-rabbit Alexa Fluor 555	Invitrogen	A31572	Polyclonal	1:500
Iba1 (chicken)	SySy	234 009	Monoclonal	1:500	Donkey anti-chicken Cy3	Sigma-Aldrich	AP194C	Polyclonal	1:400
IL-1β (rabbit)	Life Technologies	P420B	Polyclonal	1:200	Donkey anti-rabbit Alexa Fluor 488	Jackson ImmunoResearch	AB_2313584	Polyclonal	1:500
GFAP (chicken)	Millipore	AB5541	Polyclonal	1:700	Donkey anti-chicken Alexa Fluor 488	Jackson ImmunoResearch	703–545-155	Polyclonal	1:500
RBPMS (rabbit)	Millipore	ABN1362	Polyclonal	1:500	Donkey anti-rabbit Alexa Fluor 555	Millipore	AB_162543	Polyclonal	1:500
Tmem119 (rabbit)	Abcam	ab209064	Monoclonal	1:200	Donkey anti-rabbit Alexa Fluor 488	Invitrogen	A31572	Polyclonal	1:500
TNFα (goat)	Life Technologies	PA5-46945	Polyclonal	1:100	Donkey anti-goat Cy3	Abcam	ab-6949	Polyclonal	1:500

For evaluation, two central and two peripheral images from each retinal section were captured using a fluorescence microscope (Axio Imager M2; Zeiss). These images were acquired from six cross-sections per retina (24 images/eye). To ensure consistent image sizes, images were transferred to Paint Shop Pro software and excerpts were cut out in a predefined size for further evaluation (Version 13; Corel Corporation, Ottawa, Canada).

Using ImageJ software, RBPMS^+^, TNFα^+^, and cleaved caspase 3^+^ cells were counted in the GCL. Iba1^+^ microglia/macrophages and co-localized Tmem119^+^ and Iba1^+^ microglia cells were counted in the GCL+IPL+INL (GCL-INL) as well as separately in these layers. In addition, Iba1^+^ cells were evaluated in the GCL-INL based on their morphology. While the ramified cells represent their resting/surveillant state, Iba1^+^ cells with an amoeboid formation are considered as highly reactive/active (Vidal-Itriago et al., 2022; Noristani et al., 2016; Ebneter et al., 2010).

The number of CD3^+^ T-cells was evaluated by microscopy. CD3^+^ cells were counted over the entire length of the retina for each cross-section (six cross-sections per retina). The number of T-cells was evaluated in the GCL, IPL, INL, OPL, and ONL (Reinehr et al., 2024).

Evaluation of the GFAP^+^ and IL-1β^+^ areas were performed using an ImageJ macro. Initially, images were converted to grayscale. After background subtraction (Rolling Ball Radius: 50.0 pixel), the lower threshold was set to 7.86 and the upper threshold to 255 for GFAP. For IL-1β, the lower threshold was set to 22.27 and the upper threshold to 122.65. The percentage of the labeled area between these thresholds was then calculated (Palmhof et al., 2019; Hunziker et al., 2021).

### 2.6 RNA preparation and cDNA synthesis

For RNA isolation, retinas from all groups were dissected and immediately frozen at −80 °C. Two retinas from the same group were pooled for the RNA preparation and subsequent cDNA synthesis (*n* = 4 samples/group). Samples were then transferred into lysis buffer containing 2-mercaptoethanol (Sigma-Aldrich, St. Louis, MO, USA) and snap-frozen in liquid nitrogen. RNA extraction was performed using the Gene Elute Mammalian RNA Miniprep Kit (Sigma-Aldrich), followed by digestion with RNase-free DNase I (Sigma-Aldrich) (Reinehr et al., 2018; Reinehr et al., 2019). The RNA concentration was measured using Nanodrop ONE (Thermo Fisher Scientific). For reverse transcription, 1 μg of RNA was used with a cDNA synthesis kit (Thermo Fisher Scientific).

### 2.7 Quantitative real-time PCR analysis of the retina

By using a PikoReal 96 real-time PCR system (Thermo Fisher Scientific) with SYBR Green I, RT-qPCR experiments were performed (Palmhof et al., 2019; Reinehr et al., 2019). The oligonucleotides designed for RT-qPCR are listed in Table 2. *Actb* (*β*-actin) and *Ppid* (cyclophilin) served as reference genes (22). Samples that contained no cDNA but PCR grade water instead served as negative controls. Data were analyzed using REST© software (Qiagen, Hilden, Germany).

**Table 2:** List of genes used for RT-qPCR analysis.

Gene	Forward (F) and reverse (R) oligonucleotides	GenBank acc. no.
*Actb*-F *Actb*-R	CTCACCATTATATTGCTGCCTGT TCTCTTTGCCATAGCGTTTTTCT	NM_007393.5
*Bax*-F *Bax*-R	GTGAGCGGCTGCTTGTCT GTGGGGGTCCCGAAGTAG	NM_007527.3
*Bcl2*-F *Bcl2*-R	AGTACCTGAACCGGCATCTG GGGGCCATATAGTTCCACAAA	NM_009741.5
*Gfap*-F *Gfap*-R	ACAGACTTTCTCCAACCTCCAG CCTTCTGACACGGATTTGGT	NM_010277.3
*Iba1*-F *Iba1*-R	GGATTTGCAGGGAGGAAAA TGGGATCATCGAGGAATTG	D86382.1
*Il1b*-F *Il1b*-R	AGTTGACGGACCCCAAAAG AGCTGGATGCTCTCATCAGG	NM_008361.4
*Ppid*-F *Ppid*-R	TTCTTCATAACCACAGTCAAGAC TCCACCTTCCGTACCACATC	M60456.1
*Rbmps*-F *Rbmps*-R	CGCAAACGCTACGACTAGAG AGGGCTACTGGGGTAAAGTG	NM_019733.3
*Tmem119*-F *Tmem119*-R	GTGTCTAACAGGCCCCAGAA AGCCACGTGGTATCAAGGAG	NM_146162.3
*Tnfa*-F *Tnfa*-R	CTGTAGCCCACGTCGTAGC TTGAGATCCATGCCGTTG	NM_013693.3

*Actb* and *Ppid* were used as reference genes. F, forward; R, reverse; acc. no., accession number.

### 2.8 Microarray analysis of serum samples

Serum from mice of the diet and no diet groups was collected at the end of the study by heart punctation to investigate inflammatory cytokines (Reinehr et al., 2024). For each array, two blood samples were pooled to obtain the required volume (*n* = 6 samples/group). Comprehensive analyses of inflammatory protein levels were performed by using the commercially available RayBio Mouse Inflammation Antibody Array 1 (RayBiotech, Norcross, GA, USA) as described previously (Lo et al., 2010; Ray et al., 2007; Tang et al., 2012). Briefly, for each sample, one nitrocellulose membrane, containing 40 different antibodies in duplicate spots, were blocked, incubated with appropriately diluted sera (1:1), washed, and then incubated with a cocktail of biotin-conjugated antibodies specific for the different proteins. The chemiluminescent signal was detected using an imaging system (Fusion FX7 Edge; Vilber Lourmat, Eberhardzell, Germany). The resulting images were analyzed using the BIO-1D software (Vilber Lourmat) to measure the expression of various cytokines. Positive control spots, which contained a defined amount of biotinylated antibody, were used for array orientation and signal normalization. Negative control spots (buffer only) and blank spots (no printed material) were used to assess non-specific binding and background signal, respectively. For analysis, background values were subtracted using the negative control spots. The resulting data were normalized to the positive control signals to enable comparison across arrays. Furthermore, a KEGG pathway analyses was conducted using all downregulated proteins (Kanehisa et al., 2022).

### 2.9 Statistical analysis

The weight of the mice of the no diet and diet groups was analyzed using Statistica (Version 14; Dell Technologies, Round Rock, TX, USA) via two-tailed Student’s *t*-test and data are presented as mean ± standard deviation (SD) ± standard error of the mean (SEM). Immunohistological data were analyzed using Statistica with a one-way ANOVA followed by Tukey’s post-hoc test and are presented as mean ± SEM. In addition, the HSP27+diet group was compared to the HSP27 one via two-tailed Student’s *t*-test (Statistica). For RT-qPCR analyses, relative expression values are displayed as median ± quartile + minimum/maximum. Relative expression analysis was conducted using the Pair Wise Fixed Reallocation Randomization Test in REST© software (Qiagen) (Grotegut et al., 2021; Pang and Clark, 2007). For microarray analysis, no diet values were set to 100% and data are presented as mean ± SD ± SEM and the diet group was compared to the no diet group by a non-parametric Mann–Whitney U test (Statistica) (Reinehr et al., 2024; Wilmes et al., 2018). *p*-values below 0.050 were considered statistically significant, with *p < 0.050, **p < 0.010, and ***p < 0.001.

## 3 Results

### 3.1 No weight loss despite intermittent fasting

During the study period, the animals were weighed once a week to analyze if intermittent fasting contributed to weight loss. At baseline, the diet mice weight (28.13 ± 0.64 g) was analogous to the one of the no diet group (29.13 ± 1.05 g; *p* = 0.422). At week one, the weight differences between the diet (30.25 ± 0.81 g) and the no diet group (31.00 ± 1.22 g; *p* = 0.612) remained unaltered. Also, at week two, no variances were noted between the diet group (31.84 ± 0.84 g) and the no diet group (32.78 ± 1.37 g; *p* = 0.563). Moreover, no divergences were discovered in the diet group (32.59 ± 0.90 g) compared to no diet mice (33.69 ± 1.41 g; *p* = 0.518) at week 3. In the final measurement, the weight of the diet animals (34.38 ± 0.87 g) remained similar to the no diet mice (35.00 ± 1.42 g; *p* = 0.710; Supplementary Figure 1).

### 3.2 No alterations in retinal thickness

After 4 weeks, OCT images were taken of the eyes from the four groups (control, control+diet, HSP27, and HSP27+diet) to analyze possible changes in the structure of the retina (Figure 1B). The thickness of the GCC was not altered in control+diet (155.93 ± 6.29 μm; *p* = 0.992), HSP27 (164.62 ± 5.07 μm; *p* = 0.810), and HSP27+diet retinas (157.00 ± 4.95 μm; *p* = 0.999) compared to controls (158.04 ± 4.35 μm; Figure 1C). Also, no changes were noted when comparing HSP27+diet and HSP27 eyes (*p* = 0.303; Figure 1D). The total thickness was similar in control+diet (397.99 ± 16.65 μm) and control retinas (391.19 ± 5.59 μm; *p* = 0.981). Also, no changes were observed in the retinal thickness of the HSP27 (404.25 ± 9.85 μm; *p* = 0.884) and the HSP27+diet group (399.74 ± 15.31 μm; *p* = 0.963) when compared to controls (Figure 1E). In addition, the total retinal thickness was similar in HSP27+diet retinas compared to HSP27 ones (*p* = 0.809; Figure 1F).

### 3.3 Diet preserved retinal ganglion cell numbers

**Figure 2:**
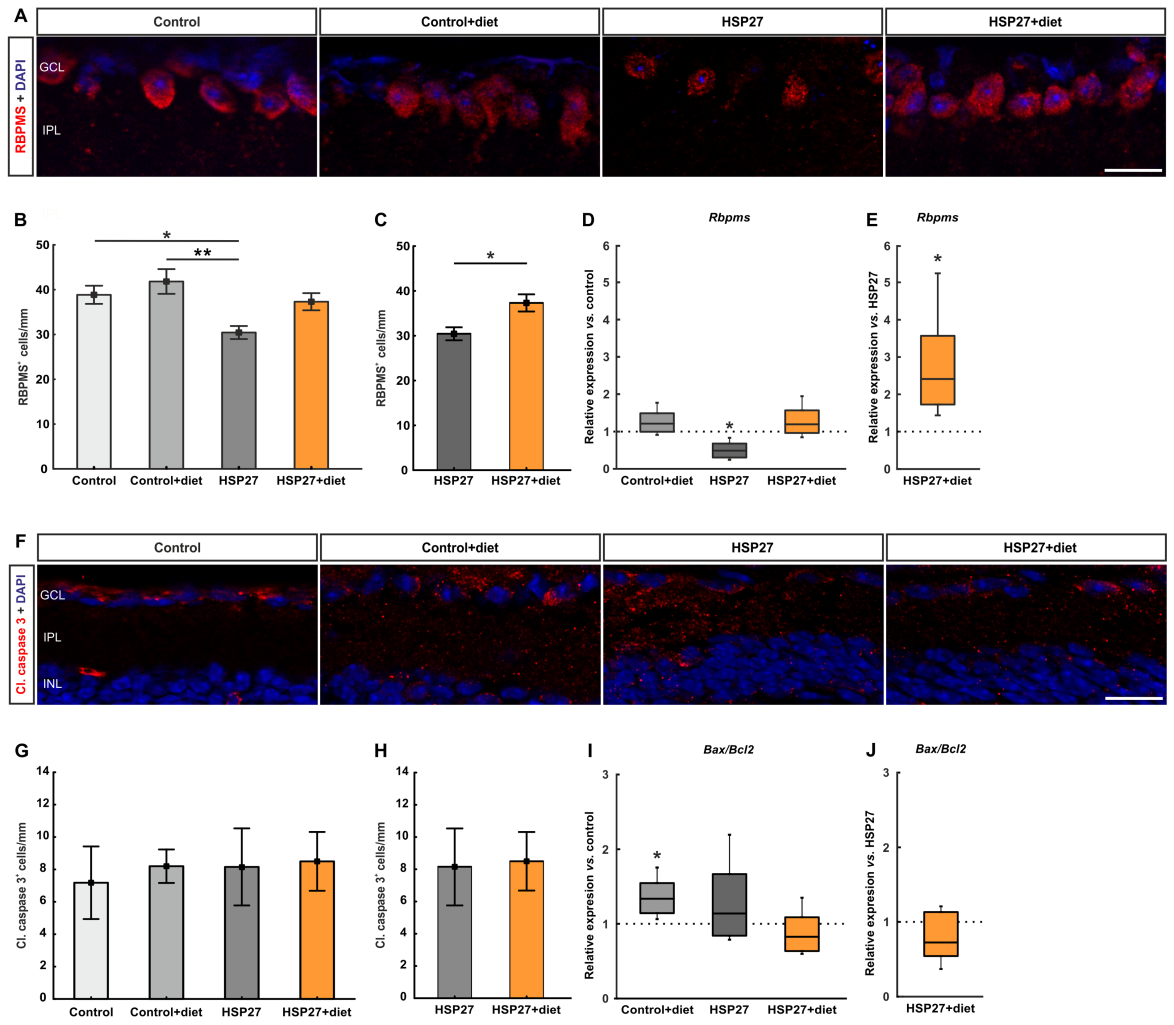
**(A)** RGCs were marked with an antibody against RBPMS (red), while the nuclei were labeled with DAPI (blue). The pictures depict representative images for each group. **(B)** Cell counts of RGCs showed a significant decrease in HSP27 group compared to the control (*p* < 0.050) and to the control+diet group (*p* < 0.010). In contrast, there was no significant change in the HSP27+diet group compared to both controls. **(C)** The RBPMS^+^ cell counts were significantly higher in HSP27+diet eyes when compared to HSP27 retinas (*p* < 0.050). **(D)** A significant downregulation of *Rbpms* was detected in the HSP27 group on mRNA level compared to the control retinas (*p* < 0.050). **(E)** In comparison to HSP27 samples, *Rbpms* was significantly upregulated in the HSP27+diet group (*p* < 0.050). **(F)** Apoptotic cells were labeled with an antibody against cleaved caspase 3 (red) and DAPI (blue) counterstained cell nuclei. The pictures depict representative images for each group. **(G)** The cell counts revealed no differences between the groups. **(H)** The number of cleaved caspase 3^+^ cells was similar in HSP27+diet and HSP27 samples. **(I)** A significant upregulation of *Bax*/*Bcl2* was noted between the control+diet group and the control group (*p* < 0.050). **(J)** No changes in *Bax*/*Bcl2* levels were noted in HSP27+diet samples compared to the HSP27 group. GCL, ganglion cell layer; IPL, inner plexiform layer, INL, inner nuclear layer. Values for immunohistology are mean ± SEM and for RT-PCR median ± quartile + minimum/maximum. Immunohistology: *n* = 6 eyes/group; RT-qPCR: *n* = 4 samples/group. The dotted lines in **(D,E)** and **(I,J)** represent the values of the respective controls. Scale bars: 20 μm. *p < 0.050 and **p < 0.010.

The loss of RGCs is a hallmark of glaucoma-like damage. Therefore, RGCs from the four groups were marked with an antibody against RBPMS by immunostaining (Figure 2A). The number of RGCs did not differ between control+diet (41.83 ± 2.76 cells/mm) and control retinas (38.85 ± 2.03 cells/mm; *p* = 0.748). A significant RGC loss was noted in the HSP27 (30.44 ± 1.45 cells/mm) compared to the control (*p* = 0.046) as well as the control+diet group (*p* = 0.005). In contrast, HSP27+diet retinas displayed no alterations in the number of RBPMS^+^ cells (37.33 ± 1.92 cells/mm) when compared to controls (*p* = 0.954; Figure 2B). Moreover, more RBPMS^+^ RGCs were observed in HSP27+diet samples when compared to HSP27 ones (*p* = 0.017; Figure 2C).

Further, RT-qPCR analyses of retinas were performed. No differences were observable in the *Rbpms* mRNA expression levels between control+diet (1.21-fold expression) and control samples (*p* = 0.272). A significant downregulation of *Rbpms* mRNA levels were observed in the HSP27 group when compared to controls (0.49-fold expression; *p* = 0.027), while no alterations were noted in HSP27+diet retinas (1.19-fold expression; *p* = 0.454, Figure 2D). In comparison to HSP27, HSP27+diet samples showed a higher *Rbpms* expression (2.41-fold expression; *p* = 0.031; Figure 2E).

Moreover, the number of apoptotic cells in the GCL was evaluated through an antibody against cleaved caspase 3 (Figure 2F). Here, the number of cleaved caspase 3^+^ cells was similar in control+diet (8.20 ± 1.03 cells/mm) and control eyes (7.18 ± 2.24 cells/mm; *p* = 0.982). Also, no difference was observed in HSP27 samples (8.16 ± 2.38 cells/mm) compared to control (*p* = 0.984) and control+diet retinas (*p* = 1.000). In addition, the HSP27+diet group (8.50 ± 1.82 cells/mm) did not differ from to the control group (*p* = 0.963; Figure 2G). Consequently, no alterations were revealed in the number of cleaved caspase 3^+^ cells between HSP27+diet and HSP27 retinas (*p* = 0.911; Figure 2H).

We also investigated the ratio of *Bax/Bcl2* using RT-qPCR. Here, a significant upregulation was noted in the control+diet group compared to the control retinas (1.34-fold-expression; *p* = 0.020). No alterations were observed in the HSP27 (1.14-fold-expression; *p* = 0.561) as well as in the HSP27+diet retinas compared (0.83-fold-expression; *p* = 0.253) to controls (Figure 2I). The *Bax*/*Bcl2* expression level remained without any alterations in the HSP27+diet samples compared to the HSP27 retinas (0.73-fold-expression; *p* = 0.172; Figure 2J).

In summary, intermittent fasting was able to preserve the number of RGCs after HSP27 injection.

### 3.4 Less macrogliosis through dieting

**Figure 3:**
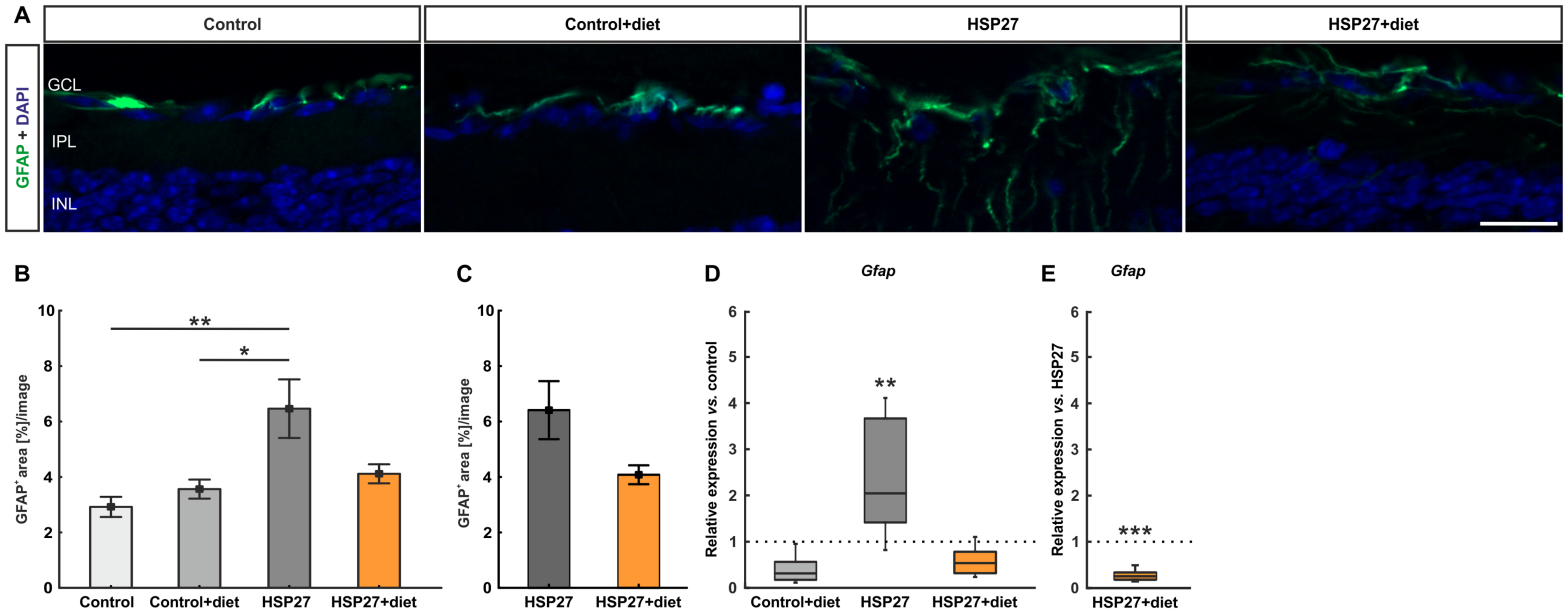
**(A)** An antibody against GFAP (green) was used to visualize the macroglia, while the nuclei were labeled with DAPI (blue). The pictures depict representative images for each group. **(B)** The GFAP^+^ area was significantly increased after HSP27 injection compared to control (*p* < 0.010) and control+diet retinas (*p* < 0.050). However, no change was detected in the HSP27+diet group compared to both controls. **(C)** The GFAP^+^ area was similiar in HSP27+diet retinas compared to the HSP27 group. **(D)** A significant upregulation of *Gfap* mRNA expression was noted in the HSP27 group compared to controls (*p* < 0.010). **(E)** A significant downregulation of *Gfap* expression was measured in the HSP27+diet group compared to the HSP27 group (*p* < 0.001). GCL, ganglion cell layer; IPL, inner plexiform layer; INL, inner nuclear layer. Values for immunohistology are mean ± SEM and for RT-PCR median ± quartile + minimum/maximum. Immunohistology: *n* = 6 eyes/group; RT-qPCR: *n* = 4 samples/group. The dotted lines in **(D,E)** represent the values of the respective controls. Scale bar: 20 μm. *p < 0.050, **p < 0.010, and ***p < 0.001.

For the analysis of macrogliosis, astrocytes in the retina were marked by immunostaining with an antibody against GFAP (Figure 3A). The area in the control+diet group (3.53 ± 0.34% area/image) did not show any difference compared to the controls (2.90 ± 0.36% area/image; *p* = 0.878). Nevertheless, the GFAP^+^ area of the HSP27 group (6.41 ± 1.05% area/image) was significantly increased compared to the control group (*p* = 0.003) and to the control+diet group (*p* = 0.015). HSP27+diet retinas (4.08 ± 0.34% area/image) demonstrated no significant differences in staining area compared to controls (*p* = 0.523; Figure 3B). No difference was noted when comparing HSP27+diet with the HSP27 group (*p* = 0.061; Figure 3C).

Complementary RT-qPCR analyses for retinas from all four groups were also performed. The expression of *Gfap* mRNA levels in the control+diet group displayed no significant difference compared to the control tissue (0.40-fold expression; *p* = 0.080). In the HSP27 group, *Gfap* expression was significantly upregulated compared to controls (2.58-fold expression; *p* = 0.001). In contrast, the HSP27+diet samples displayed no significant changes in comparison to controls (0.68-fold expression; *p* = 0.134; Figure 3D). Retinas of HSP27+diet animals revealed a significant downregulation of *Gfap* mRNA levels in comparison to HSP27 samples (0.26-fold expression; *p* < 0.001; Figure 3E).

To conclude, macrogliosis after HSP27 injection was mitigated by intermittent fasting in this normal-tension glaucoma model.

### 3.5 Lower microglia cell counts in HSP27+diet retinas

**Figure 4:**
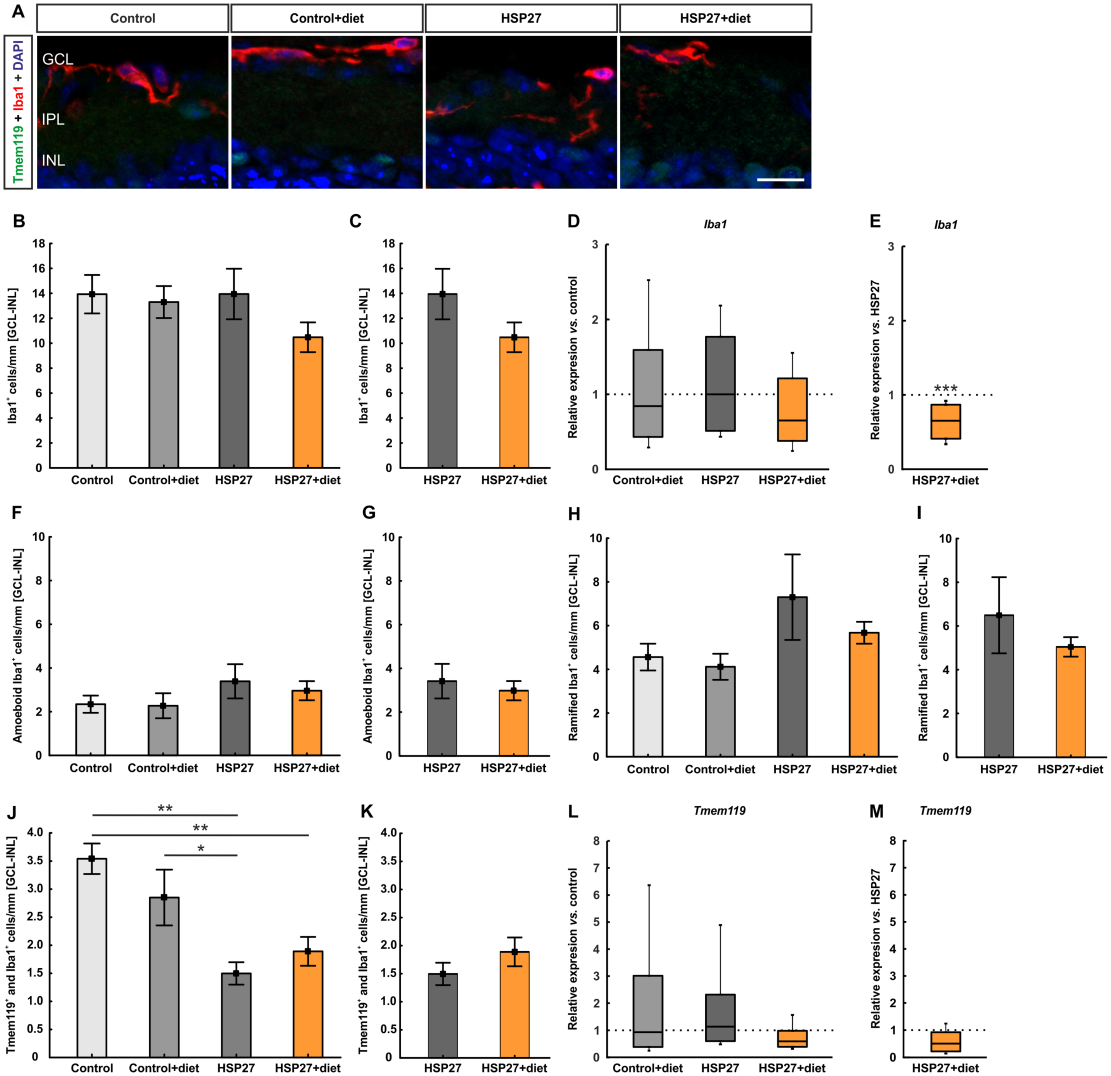
**(A)** Co-staining with antibodies against Tmem119 (green) and Iba1 (red) was performed to label microglia. Single Iba1^+^ cells display microglia/macrophages. DAPI (blue) marked the cell nuclei. The pictures depict representative images for each group. **(B)** The number of Iba1^+^ cells in the GCL-INL did not differ between all groups. **(C)** Also, no changes were observed in the HSP27+diet retinas compared to HSP27 ones. **(D)** The mRNA expression levels of *Iba1* were unaltered in all groups compared to the controls. **(E)** However, *Iba1* mRNA expression was downregulated in HSP27+diet samples when compared to the HSP27 group (*p* < 0.001). **(F)** The number of amoeboid Iba1^+^ cells was similar in all groups. **(G)** No difference was noted in Iba^+^ amoeboid cells in HSP27+diet compared to HSP27 retinas. **(H)** Similarly, the number of ramified Iba^+^ cells was comparable in all groups **(I)** Also, the number of ramified Iba1^+^ cells did not differ between HSP27+diet and HSP27 eyes. **(J)** The number of Tmem119^+^ and Iba1^+^ cells in the GCL-INL was significantly decreased in the HSP27 group compared to control (*p* < 0.010) and control+diet retinas (*p* < 0.050). Moreover, the HSP27+diet retinas displayed fewer of Tmem119^+^ and Iba1^+^ cells in the GCL-INL compared to control samples (*p* < 0.010). **(K)** The number of Tmem119^+^ and Iba1^+^ cells was similar in HSP27+diet and HSP27 retinas in the GCL-INL. **(L)** *Tmem119* mRNA expression was comparable in all groups. **(M)** Also, no alterations in *Tmem* 119 mRNA levels were detected in HSP27+diet retinas compared to HSP27 ones. GCL, ganglion cell layer; IPL, inner plexiform layer; INL, inner nuclear layer. Values for immunohistology are mean ± SEM and for RT-PCR median ± quartile + minimum/maximum. Immunohistology: *n* = 6 eyes/group; RT-qPCR: *n* = 4 samples/group. The dotted lines in **(D,E)** and **(L,M)** represent the values of the respective controls. Scale bar: 20 μm. *p < 0.050, **p < 0.010, and ***p < 0.001.

Iba1^+^ staining was used to identify microglia/macrophages, while the co-localization of Tmem119^+^ and Iba1^+^ cells was used to label retinal microglia in the GCL-INL as well as separately in each of these layers. In order to get more information on the activation state of microglia, we further differentiated Iba1^+^ cells in amoeboid and ramified cells (Figure 4A). The number of Iba1^+^ cells in the GCL-INL was similar in control+diet (13.30 ± 1.28 cells/mm; *p* = 0.991), HSP27 (13.94 ± 2.03 cells/mm; *p* = 1.000), and HSP27+diet retinas (10.48 ± 1.19 cells/mm; *p* = 0.441) compared to control ones (13.93 ± 1.54 cells/mm; Figure 4B). Congruently, no difference was noted between HSP27+diet and HSP27 eyes (*p* = 0.171; Figure 4C).

The number of Iba1^+^ microglia/macrophages in the GCL was not altered in the control+diet (8.41 ± 0.83 cells/mm) compared to control retinas (7.35 ± 1.17 cells/mm; *p* = 0.887). Moreover, the HSP27 group (9.14 ± 1.36 cells/mm) was similar compared to control eyes (*p* = 0.620). In addition, the HSP27+diet (6.10 ± 0.64 cells/mm) samples did not differ in comparison to the control group (*p* = 0.828; Supplementary Figure 2A). No difference was noted when comparing HSP27+diet and HSP27 retinas (*p* = 0.070; Supplementary Figure 2B).

The number of Iba1^+^ cells was additionally evaluated in the IPL. Differences were not seen in control+diet (1.85 ± 0.57 cells/mm; *p* = 0.717), HSP27 (1.85 ± 0.73 cells/mm; *p* = 0.712), and HSP27+diet retinas (1.13 ± 0.40 cells/mm; *p* = 0.221) compared to control ones (2.65 ± 0.37 cells/mm; Supplementary Figure 2C). Also, no changes were observed between HSP27+diet and HSP27 samples (*p* = 0.406; Supplementary Figure 2D).

In the INL, Iba1^+^ microglia/macrophages did not differ between all groups (control+diet: 3.04 ± 0.54 cells/mm, *p* = 0.751; HSP27: 2.95 ± 0.74 cells/mm, *p* = 0.688; HSP27+diet: 3.25 ± 0.60 cells/mm, *p* = 0.868) compared to the control samples (3.93 ± 0.60 cells/mm; Supplementary Figure 2E). Further, Iba1^+^ cells in the INL were comparable in the HSP27+diet and HSP27 retinas (*p* = 0.757; Supplementary Figure 2F).

RT-qPCR analyses of the *Iba1* mRNA expression revealed no alterations in the control+diet samples in comparison to controls (0.85-fold expression; *p* = 0.619). Also, no changes were noted in HSP27 (1.00-fold expression; *p* = 0.959) as well as in HSP27+diet samples (0.65-fold expression; *p* = 0.279) compared to controls (Figure 4D). Interestingly, a significant *Iba1* downregulation was observed for the HSP27 retinas (0.65-fold expression) compared to the HSP27+diet samples (*p* < 0.001; Figure 4E).

We further differentiated Iba1^+^ cells in amoeboid and ramified ones. Here, the number of amoeboid Iba1^+^ cells in the GCL-INL did not differ in control+diet (2.27 ± 0.57 cells/mm; *p* = 1.000), HSP27 (3.39 ± 0.78 cells/mm; *p* = 0.570), and HSP27+diet retinas (2.96 ± 0.44 cells/mm; *p* = 0.867) compared to control ones (2.34 ± 0.40 cells/mm; Figure 4F). Accordingly, no changes could be noted between HSP27+diet and HSP27 samples (*p* = 0.641; Figure 4G). Moreover, the number of ramified Iba1^+^ cells was examined in the GCL-INL. No difference was noted between control+diet (4.07 ± 0.59 cells/mm; *p* = 0.992), HSP27 (7.22 ± 1.94 cells/mm; *p* = 0.317), and HSP27+diet eyes (5.61 ± 0.50 cells/mm; *p* = 0.889) compared to the control group (4.51 ± 0.61 cells/mm; Figure 4H). Also, the number of ramified Iba1^+^ cells was similar in HSP27+diet and HSP27 retinas (*p* = 0.439; Figure 4I).

In the GCL-INL, the number of Tmem119^+^ and Iba1^+^ microglia cells was not altered between control+diet (2.85 ± 0.50 cells/mm) and control retinas (3.54 ± 0.27 cells/mm; *p* = 0.458). However, a lower cell number was noted in HSP27 samples (1.50 ± 0.20 cells/mm) compared to control (*p* = 0.002) and control+diet retinas (*p* = 0.040). Also, lower microglia numbers were detected in HSP27+diet samples (1.89 ± 0.26 cells/mm) compared to the control eyes (*p* = 0.010; Figure 4J). No differences were revealed between HSP27+diet and HSP27 retinas (*p* = 0.255; Figure 4K).

No alteration in the number of Tmem119^+^ and Iba1^+^ cells in the GCL was observed between the control+diet (1.05 ± 0.14 cells/mm; *p* = 0.626) as well as HSP27 retinas (0.82 ± 0.20 cells/mm; *p* = 0.187) compared to controls (1.37 ± 0.23 cells/mm). In contrast, microglia numbers in the HSP27+diet group (0.38 ± 0.16 cells/mm) were significantly decreased compared to the control situation (*p* = 0.006; Supplementary Figure 2G). No changes were observed between HSP27+diet and HSP27 samples (*p* = 0.111; Supplementary Figure 2H).

The number of Tmem119^+^ and Iba1^+^ cells in the IPL was not altered in control+diet (0.72 ± 0.23 cells/mm; *p* = 0.877), HSP27 (0.28 ± 0.06 cells/mm; *p* = 0.711), and HSP27+diet retinas (0.59 ± 0.19 cells/mm; *p* = 0.997) compared to control ones (0.54 ± 0.16 cells/mm; Supplementary Figure 2I). Also, no changes were seen between HSP27+diet and HSP27 samples (*p* = 0.149; Supplementary Figure 2J).

In the INL, Tmem119^+^ and Iba1^+^ microglia did not differ between control+diet (1.07 ± 0.20 cells/mm) and control eyes (1.63 ± 0.22 cells/mm; *p* = 0.194). The number of Tmem119^+^ and Iba1^+^ cells was significantly lower in HSP27 retinas (0.39 ± 0.13 cells/mm) compared to the control situation (*p* = 0.001). No alteration was observed between the HSP27+diet (0.92 ± 0.20 cells/mm) and control retinas (*p* = 0.069; Supplementary Figure 2K). The number of Tmem119^+^ and Iba1^+^ cells was comparable in the HSP27+diet and HSP27 retinas (*p* = 0.052; Supplementary Figure 2L).

For *Tmem119* mRNA levels, no difference between control+diet (0.93-fold expression) and control retinas was noted (*p* = 0.926). Also, no alteration was observed for HSP27 (1.13-fold expression; *p* = 0.826) and HSP27+diet samples (0.59-fold expression; *p* = 0.085) compared to control tissue (Figure 4L). Furthermore, HSP27+diet retinas showed no significant change in *Tmem119* mRNA expression compared to HSP27 tissue (0.52-fold expression; *p* = 0.077; Figure 4M).

In summary, in HSP27+diet retinas a lower number of Tmem119^+^ and Iba1^+^ microglia could be noted.

### 3.6 Decreased inflammatory response after fasting

**Figure 5:**
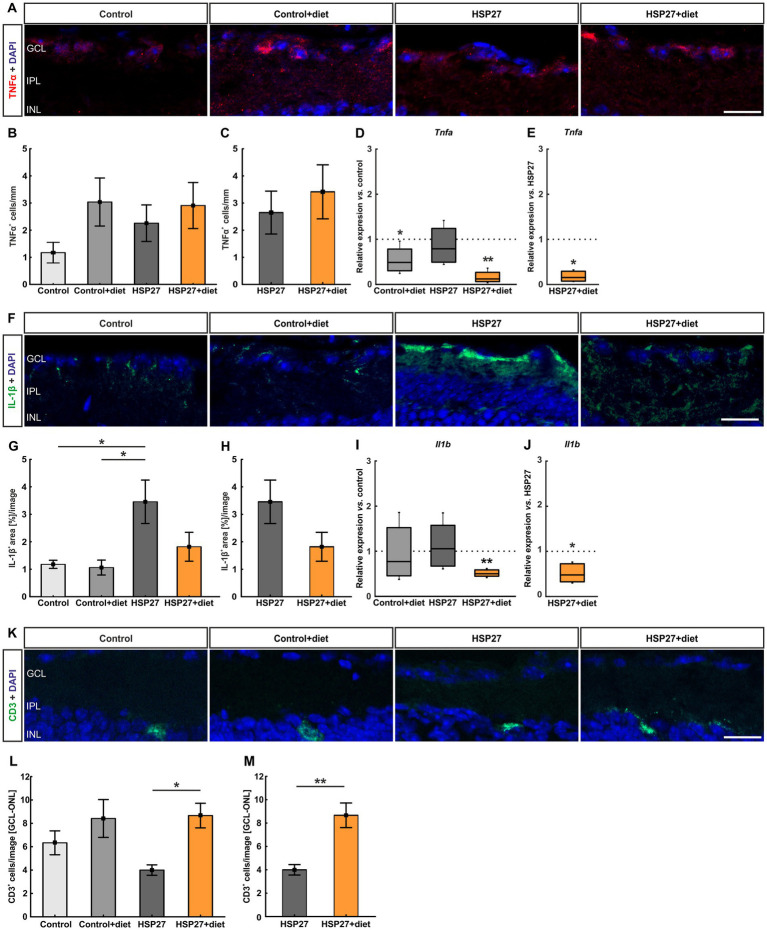
**(A)** Retinal cross-sections were stained with an antibody against TNF*α*^+^ (red) and cell nuclei were labeled with DAPI (blue). The pictures depict representative images for each group. **(B)** The number of TNFα^+^ cells was similar within all groups. **(C)** Also, no changes were observed in the HSP27+diet retinas compared to HSP27 ones. **(D)** A significant downregulation of *Tnfa* mRNA expression levels was observed in the control+diet (*p* < 0.050) as well as in the HSP27+diet samples (*p* < 0.010). **(E)** Moreover, the expression of *Tnfa* in HSP27+diet retinas was significantly reduced compared to the HSP27 group (*p* < 0.050). **(F)** Further, an antibody against IL-1β antibody (green) was used, while the nuclei were marked by DAPI (blue). The pictures depict representative images for each group. **(G)** The IL-1β^+^ area of HSP27 retinas was significantly larger in comparison with both controls (*p* < 0.050), while no changes could be noted in HSP27+diet retinas. **(H)** The HSP27+diet group did not differ compared to the HSP27 retinas regarding the IL-1β^+^ area. **(I)** A significant downregulation of *Il1b* expression levels was detected in HSP27+diet retinas compared to control samples (*p* < 0.010). **(J)** Also, *Il1b* expression levels were downregulated in HSP27+diet tissue when compared to the HSP27 group (*p* < 0.050). **(K)** Retinas of all groups were labeled with an antibody against CD3 to detect pan T-cells (green), while DAPI (blue) counterstained cell nuclei. The pictures depict representative images for each group. **(L)** The number of CD3^+^ cells in the GCL-ONL was significantly higher in the HSP27+diet group than in HSP27 retinas (*p* < 0.050). **(M)** A higher number of CD3^+^ cells was revealed in HSP27+diet retinas compared to the HSP27 group (*p* < 0.010; GCL-ONL). GCL, ganglion cell layer; IPL, inner plexiform layer; INL, inner nuclear layer. Values for immunohistology are mean ± SEM and median ± quartile + minimum/maximum for RT-PCR. Immunohistology: *n* = 6 eyes/group; RT-qPCR: *n* = 4 samples/group. The dotted lines in **(D,E)** and **(I,J)** represent the values of the respective controls. Scale bars: 20 μm. *p < 0.050 and **p < 0.010.

To assess the inflammatory response after HSP27 injection and dietary effects, retinal cross-sections were stained with antibodies against TNFα and IL-1β (Figures 5A,F). Regarding the number of TNFα^+^ cells, no alteration was observed between control+diet (3.04 ± 0.89 cells/mm) and control retinas (1.17 ± 0.38 cells/mm; *p* = 0.293). The HSP27 group (2.26 ± 0.67 cells/mm) also showed no changes in cell numbers compared to the control (*p* = 0.717) or the control+diet group (*p* = 0.871). Furthermore, TNFα^+^ cell counts did not differ between HSP27+diet (2.91 ± 0.85 cells/mm) and control tissue (*p* = 0.352; Figure 5B). Also, no changes were observed when comparing HSP27+diet with HSP27 retinas (*p* = 0.561; Figure 5C).

Interestingly, the mRNA expression levels of *Tnfa* were significantly downregulated in control+diet samples (0.49-fold expression) compared to control retinas (*p* = 0.041). In HSP27 samples, the mRNA levels of *Tnfa* were not altered (0.79-fold expression; *p* = 0.324), while in HSP27+diet retinas a significant downregulation was observed (0.12-fold expression; *p* = 0.004; Figure 5D). Moreover, the HSP27+diet group had downregulated *Tnfa* levels when compared to the HSP27 group (0.16-fold expression; *p* = 0.013; Figure 5E).

The IL-1β^+^ staining area of the control+diet group (1.06 ± 0.27% area/image) was similar to the control situation (1.18 ± 0.15% area/image; *p* = 0.998). However, the IL-1β^+^ area of the HSP27 group (3.46 ± 0.79% area/image) was larger compared to both control (*p* = 0.021) and control+diet samples (*p* = 0.014). In comparison to the controls, there was no difference in the HSP27+diet group (1.82 ± 0.53% area/image; *p* = 0.802; Figure 5G). No alterations were noted in the HSP27+diet retinas compared to the HSP27 ones (*p* = 0.116; Figure 5H).

Also, *Il1b* mRNA expression did not differ between the control+diet samples and controls (0.77-fold expression; *p* = 0.410). In addition, HSP27 retinas displayed no alteration in comparison to the controls (1.06-fold expression; *p* = 0.822). However, there was a significant *Il1b* downregulation in the HSP27+diet group compared to the control group (0.50-fold-expression; *p* = 0.007; Figure 5I). A downregulated *Il1b* expression in the HSP27+diet group was also found in comparison to HSP27 retinas (0.48-fold expression; *p* = 0.018; Figure 5J).

In conclusion, intermittent fasting was able to reduce retinal inflammation after intravitreal HSP27 injection.

### 3.7 Increased T-cell response in HSP27+diet retinas

To evaluate the T-cell response in the retina, cross-sections were labeled with an antibody against CD3 (Figure 5K). There was no significant difference in the CD3^+^ cell number between the control+diet retinas (8.42 ± 1.62 cells/image) and the controls (6.33 ± 1.02 cells/image; *p* = 0.561). Regarding the HSP27 group (4.00 ± 0.45 cells/image), no alteration was observed compared to the control (*p* = 0.468) and the control+diet group (*p* = 0.050). T-cell counts in HSP27+diet samples (8.67 ± 1.05 cells/image) were similar to controls (*p* = 0.468). However, significantly more CD3^+^ cells were noted in the HSP27+diet retinas compared to the HSP27 group (*p* = 0.036; Figure 5L). This was also the case when comparing the HSP27+diet with the HSP27 group (*p* = 0.002; Figure 5M).

### 3.8 Lower pro-inflammatory cytokine levels in diet serum samples

**Figure 6:**
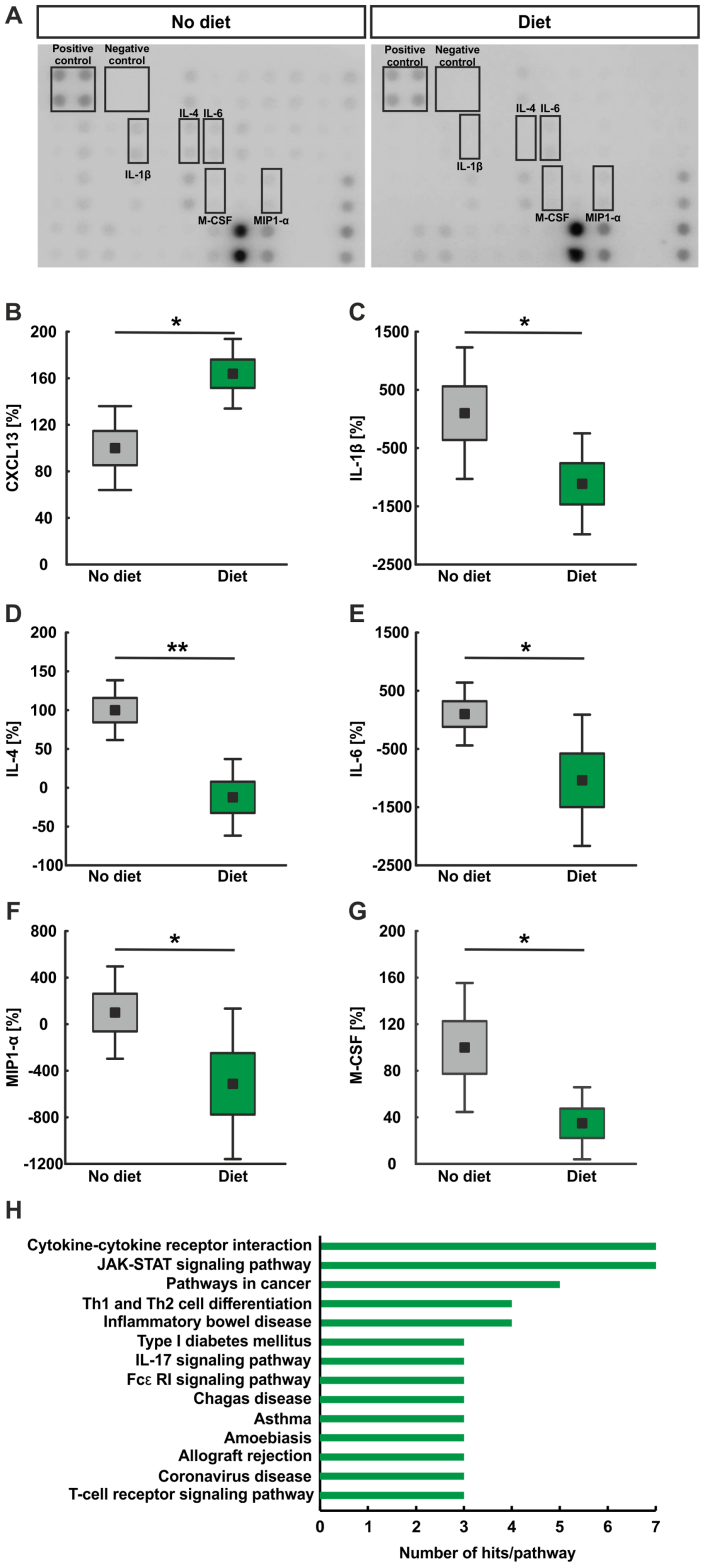
**(A)** Exemplary microarray pattern after incubation with serum samples from mice that had no diet or were dieting. **(B)** CXCL13 levels were significantly upregulated in diet serum samples compared to no diet ones (*p* < 0.050). **(C–E)** The serum levels of IL-1β (*p* < 0.050), IL-4 (*p* < 0.010), and IL-6 (*p* < 0.050), were significantly lower in diet serum samples when compared to no diet ones. **(F)** MIP1-α was decreased in diet samples compared to no diet ones (*p* < 0.050). **(G)** In addition, lower M-CSF levels were measured in diet samples compared to no diet ones (*p* < 0.050). **(H)** Overview of the KEGG analyses. Values are mean ± SEM ± SD. *p < 0.050 and **p < 0.010.

For microarray assays, we used serum samples of mice, who either had food *ad libitum* (no diet) or underwent intermitted fasting (diet; Figure 6A). In total, the intensity of 40 proteins was detected (Supplementary Table 1) and the six most interesting and regulated ones are presented here. Regarding the CXC-motif chemokine 13 (CXCL13), a significantly higher percentage was noted in the diet serum samples (163.89 ± 12.22%) compared to the no diet group (100.00 ± 14.71%; *p* = 0.020; Figure 6B). In contrast, for IL-1β, we observed significantly lower serum levels in the diet group (−1,113.83 ± 354.04%) than in the no diet group (100.00 ± 461.21%; *p* = 0.031; Figure 6C). Also, IL-4 serum levels were significantly lower in diet animals (−12.35 ± 20.16%) when compared to no diet mice (100.00 ± 15.74%; *p* = 0.008; Figure 6D). IL-6 levels were also significantly lower in the diet group (−1,038.15 ± 460.42%) compared to the no diet serum samples (100.00 ± 220.42%; *p* = 0.031; Figure 5E). Moreover, the serum levels of the macrophage inflammatory protein 1α in the diet serum (MIP-1α; −512.18 ± 263.85%) was lower as in no diet mice (100.00 ± 161.97%; *p* = 0.045; Figure 6F). Also, the macrophage colony-stimulating factor (M-CSF) levels were found to be significantly lower in diet mice (34.89 ± 12.63%) compared to no diet ones (100.00 ± 22.63%; *p* = 0.045; Figure 6G). Moreover, eight additional proteins were significantly regulated. While IL-2 was found upregulated in diet mice (*p* = 0.045), GM-CSF (*p* = 0.031), IFN-*γ* (*p* = 0.045), IL-3 (*p* = 0.045), IL-9 (*p* = 0.045), IL-12 p70 (*p* = 0.005), IL-13 (*p* = 0.045), and MIP-1γ (*p* = 0.008) were decreased in diet animals (Supplementary Table 1). The KEGG analyses revealed that all seven downregulated proteins were associated with the cytokine-cytokine receptor interaction and the JAK–STAT signaling pathway. Five proteins were linked with Th1 and Th2 cell differentiation and inflammatory bowel disease. Three proteins were connected to several pathways, including IL-17, Fcε R1, and T-cell receptor signaling (Figure 6H). All pathways from the analyses are shown in Supplementary Table 2.

## 4 Discussion

Neurodegenerative diseases pose a major public health challenge and still lack effective preventive and disease-modifying therapies. For glaucoma, the only approved therapy is lowering the intraocular pressure. This may slow down the progression but does not cure these patients (Lusthaus and Goldberg, 2019; Schuster et al., 2020). Despite novel therapeutic approaches, recent research has focused on the investigation of non-pharmacological methods that might also be effective in mitigating the progression of glaucoma. For example, several studies have explored the relationship between neurodegeneration and caloric restriction. The ability of RGCs to regenerate is strongly age-dependent but can potentially be influenced by therapeutic intervention. Studies in animal models have shown that restricting calorie intake by 40–60% increases lifespan by 30–50% and reduces the incidence of age-related diseases, including neurodegeneration (Omodei and Fontana, 2011; Mattison et al., 2017).

Therefore, the study presented here aimed to investigate the influence of diet on pressure-independent, immune-induced glaucoma damage for the first time. Previous studies revealed that an intravitreal injection of HSP27 leads to glaucoma-like damage in rats and mice (Grotegut et al., 2020; Erb et al., 2023). In general, HSP27 exhibits a dual capacity to modulate inflammation through its differential effects on monocyte and macrophage subsets, which largely depend on its localization (intracellular or extracellular) and the activation state of the immune microenvironment. Intracellular HSP27 acts as a cytoprotective chaperone that stabilizes the cytoskeleton, limits oxidative stress, and negatively regulates pro-inflammatory signaling cascades, including the NF-κB pathway (Lindquist and Craig, 1988; Ruan et al., 2015). This activity favors an anti-inflammatory phenotype, characterized by enhanced IL-10 production, increased M-CSF secretion, and differentiation toward macrophage subsets with reduced antigen-presenting capacity and attenuated T-cell stimulation (Hadadi et al., 2016; Miller-Graziano et al., 2008). Conversely, extracellular HSP27, released during cellular stress or necrosis, functions as a damage-associated molecular pattern that can engage toll-like receptor (TLR)2 and TLR4 on monocytes and macrophages. This can lead to NF-κB activation and the production of both pro-inflammatory and regulatory cytokines (Salari et al., 2013; Jin et al., 2014). Thus, the duality of HSP27 likely reflects a physiological mechanism to balance immune activation and resolution.

In the current study, half of the mice were subject to intermittent fasting (diet group) after intravitreal HSP27 injection. More precisely, access to food was restricted on Mondays, Wednesdays, and Fridays for 24 h (Kong et al., 2012). After weekly weigh-ins, final analyses were performed 4 weeks after HSP27 injection. The first thing we noticed was that there was no weight difference between diet and no diet mice. This aligns with a study by Anson et al. (2003), where intermittent fasting did not lead to significant weight differences in C57/BL6 mice up to 20 weeks. Especially within the first five weeks, fasting and control animals weighed the same. Intermittent fasting for six months, on the other hand, lead to a significant weight loss in C57/BL6 mice (Kong et al., 2012). Thus, food restrictions every other day for 28 days were not sufficient to lose weight. We assume that mice in the diet group ate more on the days where chow was available. Although we did not measure the food intake, similar effects were shown previously. Here, C57BL/6 mice subjected to intermittent fasting were consuming essentially the same amount of food in a 48 h period as those mice fed chow *ad libitum*. The authors stated that on the days mice had access to food, they ate roughly twice as much as the controls (Anson et al., 2003).

Although intermittent fasting did not influence body weight, it exerted a protective effect on RGCs in our study. In detail, we noted that HSP27 injection significantly reduced RBPMS^+^ cell numbers and led to a downregulation of *Rbmps* mRNA levels. In contrast, the HSP27 group undergoing the diet revealed a similar number of RGCs as well as comparable mRNA levels as controls, suggesting a protective effect of intermittent fasting on these neurons. This is comparable to mice, which lack the glutamate transport EAAC1. The same dietary regime as in our study protected RGCs compared to no diet mice. Moreover, an improvement in visual function was observed in the mentioned study. This was evidenced by enhanced electrophysiological responses in multifocal electroretinography (Guo et al., 2016). Furthermore, RGCs were preserved in ischemia/reperfusion injury in young (2-month-old) as well as old (2-year-old) rats after 3 months of caloric restriction (Kawai et al., 2001). Already 48 h of fasting significantly reduced the RGC loss induced by acute ocular hypertension (Russo et al., 2018).

Although the exact mechanisms of RGCs degeneration are not fully understood yet, it is well established that apoptosis is the earliest form of cell loss in glaucoma (Galvao et al., 2013; Xia and Zhang, 2024). In our study, the number of cleaved caspase 3^+^ cells was similar in all groups, indicating that neither HSP27 injection nor diet influenced apoptotic cells at this time point. Further, the mRNA expression ratio of *Bax*/*Bcl2* was neither altered in HSP27 nor in HSP27+diet retinas. In a streptozoticin-induced diabetes model in mice, intermitting fasting (food deprivation for 24 h, every other day for 4 weeks), the protein levels of cleaved caspase 3 and Bax/Bcl2 were decreased compared to control diabetic mice (Xiong et al., 2023). After traumatic brain injury in rats, an increased Bcl2/Bax mRNA and protein ration was noted, which reflects a shift to a pro-survival state of cells (Loncarevic-Vasiljkovic et al., 2016). The rats underwent dietary restriction 3 months prior to the injury, while our study only lasted for 28 days. Further, we injected HSP27 at the start of the diet regime. This might lead to the results regarding the apoptosis in our study. Intravitreal injection of HSP27 in rats resulted in upregulated *Casp3* mRNA levels (Grotegut et al., 2021). This could be noted 14 days after application, indicating that our examined time point might be too late to detect alterations in apoptosis. However, subsequent studies are needed to investigate the role of pro- and anti-survival apoptosis genes and proteins.

In glaucoma, neuroinflammatory processes are often observed at early stages of the disease, such as macro- and microglia activation and enhanced pro-inflammatory cytokines (Soto and Howell, 2014; Johnson and Morrison, 2009). In the HSP27-induced glaucoma model, the number of microglia/macrophages did not differ, while an upregulated *Iba1* and *Tmem119* mRNA expression was noted previously (Erb et al., 2023). Also in our study, HSP27 injection itself did not affect the number or the activation state of microglia cells. However, fewer microglia were noted in HSP27+diet eyes, suggesting that the dietary regime was able to reduce the inflammatory response in general. Similar results were noted after a traumatic brain injury. The authors demonstrated that caloric restriction prior to the injury suppresses microglial activation (Loncarevic-Vasiljkovic et al., 2016). In general, microglial activation often precedes glaucomatous damage (Noristani et al., 2016; Bosco et al., 2011; Bordone et al., 2017; Yu et al., 2025). Therefore, our time point was probably beyond the period of active microglial response. A shorter period should be included in a subsequent study to investigate the influence of intermitting fasting on microglia cells in more detail.

It is known that microglia interact with T-cells (Goldmann and Prinz, 2013). In humans, an imbalance between Th1 and Th2 T-cells contributes to a pro-inflammatory environment. In eyes from glaucoma donors, CD3^+^ T-cells have been detected (Gramlich et al., 2013; Wong et al., 2015; Guo et al., 2018). A study by Saini et al. (2023) demonstrated that Th1 cells targeting HSP27, HSP60, and *α*-crystallin were significantly more prevalent in peripheral blood mononuclear cells of patients with primary open-angle glaucoma compared to controls. Elevated levels of these HSP-specific Th1 cells were linked to a thinner retinal nerve fiber layer in affected individuals. This study builds upon earlier animal research, where a T-cell response specific to HSP27 was identified in a mouse model of ocular hypertension (Chen et al., 2018). Interestingly, we observed the lowest number of CD3^+^ T-cells in HSP27 retinas, while most T-cells were detected in both control+diet and HSP27+diet samples. In other studies, it was previously shown that fasting reshapes T-cell metabolism. For instance, dietary restriction helps regulate T-cell energy balance by enhancing glucocorticoid production and suppressing glycolysis (Collins et al., 2019; Palma et al., 2021). Furthermore, in aged mice, dietary restriction enhanced the preservation of naïve T-cells and helped maintain the diversity of the T-cell receptor repertoire, while also preventing declines in antigen presentation and T-cell proliferation (Yang et al., 2009). In tilapia, short-term fasting led to increased size of T-cells and CD3 protein expression (Li et al., 2023). The authors suggest that fasting might induce T-cell activation, to some extent, in the absence of antigen stimulation. In our study, we solely counted CD3^+^ T-cells in retinal cross-sections. Future investigations should adopt more comprehensive approaches, such as flow cytometry, to better characterize T-cell populations. Additionally, examining distinct T-cell subtypes will be important to determine whether fasting promotes a shift toward a protective phenotype.

The benefits of caloric restriction are thought to involve reduced oxidative stress and inflammation (Madeo et al., 2019). We therefore examined pro-inflammatory markers in the retina as well as in the serum. It was evident that intermittent fasting significantly reduced those investigated cytokines. Although the number of TNFα^+^ cells was similar in all groups, lower *Tnfa* mRNA expression levels were noted in control+diet as well as HSP27+diet retinas. TNFα is one of the cytokines produced by microglia cells. In aqueous humor and serum samples from patients with primary open-angle glaucoma, enhanced TNFα levels were observed (Kondkar et al., 2018; Alapati et al., 2021; Sawada et al., 2010; Balaiya et al., 2011). In our study, intermittent fasting reduced *Tnfa* levels in control and HSP27 retinas. This aligns with a study in healthy humans, where caloric restriction also decreased the levels of circulating TNFα (Ravussin et al., 2015). IL-1β is another pro-inflammatory cytokine and is produced as an inactive precursor called pro-IL-1β mainly by inflammatory cells of myeloid lineage. Pro-IL-1β is rapidly induced upon exposure of inflammatory cells to pathogen-associated molecular patterns or damage-associated molecular patterns that bind to pattern recognition receptors to upregulate pro-inflammatory gene expression. For example, the activation of inflammasomes causes a process to mature the pro-IL-1β into a form that can be secreted (Martín-Sánchez et al., 2016). In the normal-tension experimental autoimmune glaucoma model, we previously observed enhanced levels of IL-1β in the aqueous humor of rats (Reinehr et al., 2018). Upregulated *Il1b* mRNA levels were also noted after intravitreal HSP27 injection (Erb et al., 2023). This was confirmed by our study, where we not only found upregulated *Il1b* mRNA levels, but also an increased IL-1β^+^ staining area in HSP27 injected eyes. Nonetheless, the HSP27+diet group showed no changes in the IL-1β^+^ staining area. Moreover, the mRNA expression levels of *Il1b* were significantly downregulated in the HSP27+diet retinas compared to control as well as HSP27 retinas. The additional microarray analyses revealed lower IL-1β serum levels in mice who underwent intermittent fasting. Also, the serum levels of IL-4, IL-6, MIP-1*α*, and M-CSF were decreased in diet mice. In obese humans with metabolic syndrome, a balanced hypocaloric diet for 6 months resulted in weight loss accompanied by a reduction in serum pro-inflammatory cytokines such as IL-6, TNFα, IL-8, and MIP-1β (Montefusco et al., 2021). A recent cross-sectional study examined the impact of diet, sleep, and metabolomic pathways on glaucoma and found a pivotal role of balanced diets and optimized sleep patterns in glaucoma prevention and management (Shengnan et al., 2025). Altogether, our results suggest that non-pharmacological interventions like intermittent fasting could meaningfully complement existing glaucoma therapies.

We recognize that fasting is associated with autophagy, a cellular pathway that removes damaged proteins and organelles and supports metabolic resilience (Bagherniya et al., 2018). Autophagy plays a dual role in the health of RGCs. Under physiological or moderate stress conditions, it helps clear damaged organelles and proteins, supporting RGC survival. In contrast, dysregulated or excessive autophagy can contribute to RGC death (Adornetto et al., 2020). In various experimental glaucoma and optic nerve injury models, altered autophagy markers (e.g., LC3, p62, or mitophagy regulators) and impaired autophagic flux have been associated with progressive RGC loss. It is known that the balance of upstream regulators, including mTOR, AMPK, and mitophagy-related pathways, determines whether autophagy is protective or deleterious. The modulation of autophagy may represent a promising strategy to preserve RGCs in glaucomatous degeneration (Russo et al., 2015; Lee et al., 2021). For example, recent work has highlighted the potential neuroprotective effects of autophagy-modulating interventions in glaucoma. It was demonstrated that metformin protects RGCs in a murine model of retinal ischemia/reperfusion, accompanied by increased autophagy and mitophagy signaling. In addition, diabetic glaucoma patients receiving metformin exhibited greater stability of visual fields over 6 months compared with insulin-treated controls. These findings support metformin as a neuroprotective agent acting through metabolic and autophagy-related pathways and thereby simulating fasting (Satriano et al., 2025; Cuyas et al., 2019). A recent epidemiological analysis examined the effect of intermittent fasting (repeated breakfast skipping) and glaucoma. In this retrospective cross-sectional study, the results yielded to no significant association with glaucoma prevalence and fasting. However, it must be noted that the data is based on self-reporting of the participants, and that skipping breakfast is not completely considered intermitting fasting (Schulz et al., 2025). Together, these findings indicate that while metabolic or pharmacologic induction of autophagy may hold therapeutic promise, carefully controlled mechanistic and clinical studies are required to determine how best to harness autophagy for glaucoma neuroprotection.

We acknowledge that our study has some limitations. First, the utilized HSP27 model captures only one aspect of glaucoma pathogenesis, specifically immune-mediated RGC loss, and does not involve elevated intraocular pressure. Therefore, the findings should be interpreted in the context of immune-mediated damage. We did not determine which specific region of the HSP27 protein mediates the observed effects. In the present study, we used recombinant full-length HSP27. Therefore, the responses we observed most likely reflect the integrated activity of the intact protein, including contributions from its α-crystallin domain and its ability to form oligomers. Previous studies suggest that extracellular HSP27 signaling relies on recognition of its quaternary structure by innate immune receptors such as TLR2 and TLR4 rather than on a single peptide motif. Defining the minimal active domain of HSP27 would require targeted domain-mapping or truncation approaches, which represent important directions for future work. Furthermore, we only examined one time point in this study. Extending the analysis to earlier (1–2 weeks) or later (8–12 weeks) time points would certainly provide valuable insight into the kinetics of immune priming and neurodegeneration. Moreover, subsequent studies should incorporate targeted functional assessments of RGC activity, such as pattern electroretinogram or visual evoked potentials, to confirm whether the molecular and structural preservation observed under intermittent fasting translates into measurable visual benefits. Further, the lack of Western blot analysis is a limitation of the present study. Although limited tissue availability prevented us from conducting this experiment, future work with adequate material for both molecular and protein-level validation will be important to reinforce and expand our findings.

## 5 Conclusion

In summary, our findings highlight the neuroprotective and anti-inflammatory potential of intermittent fasting in a pressure-independent, immune-mediated glaucoma model. Despite no significant weight loss, dietary restriction effectively preserved RGCs and reduced both retinal and systemic pro-inflammatory cytokine levels. While the exact molecular mechanisms remain elucidated, modulation of neuroinflammation appears to play a key role. Collectively, these data support growing evidence that metabolic and lifestyle factors may critically influence the progression and management of neurodegenerative eye diseases like glaucoma.

## Data Availability

The raw data supporting the conclusions of this article will be made available by the authors, without undue reservation.
