# Supplementary material for: Intermittent fasting reduces glaucomatous damage in an HSP27 autoimmune mouse model
Source: Front Cell Neurosci. 2026 Jan 20;19:1690991. doi: 10.3389/fncel.2025.1690991 (PMC12864140; doi:10.3389/fncel.2025.1690991)
Supplement: Supplementary file 1 [file Data_Sheet_1.pdf]

frontiers

Supplementary Material

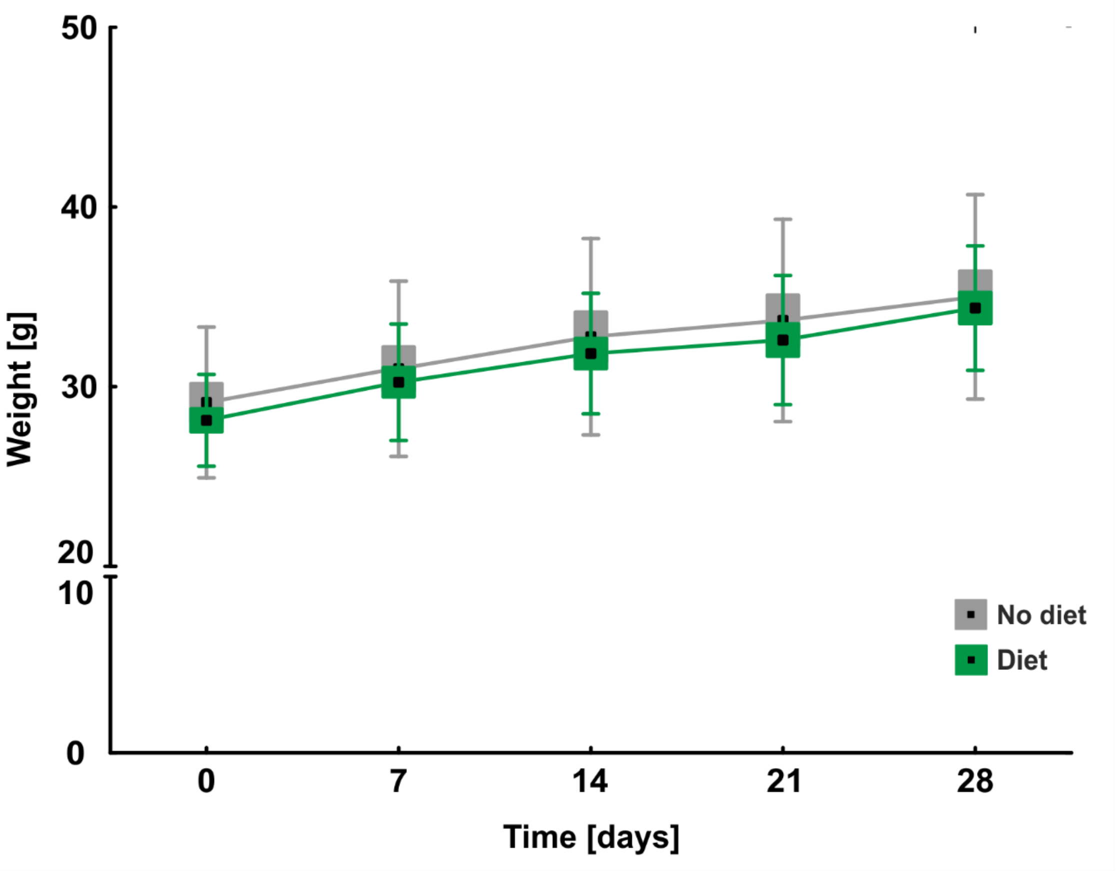

Supplementary Figure 1. No weight loss despite intermittent fasting. All mice were weighed weekly for the duration of four weeks. There was no significant difference in weight at any time point between the diet (green) and the no diet group (grey). Values are mean±SEM±SD.

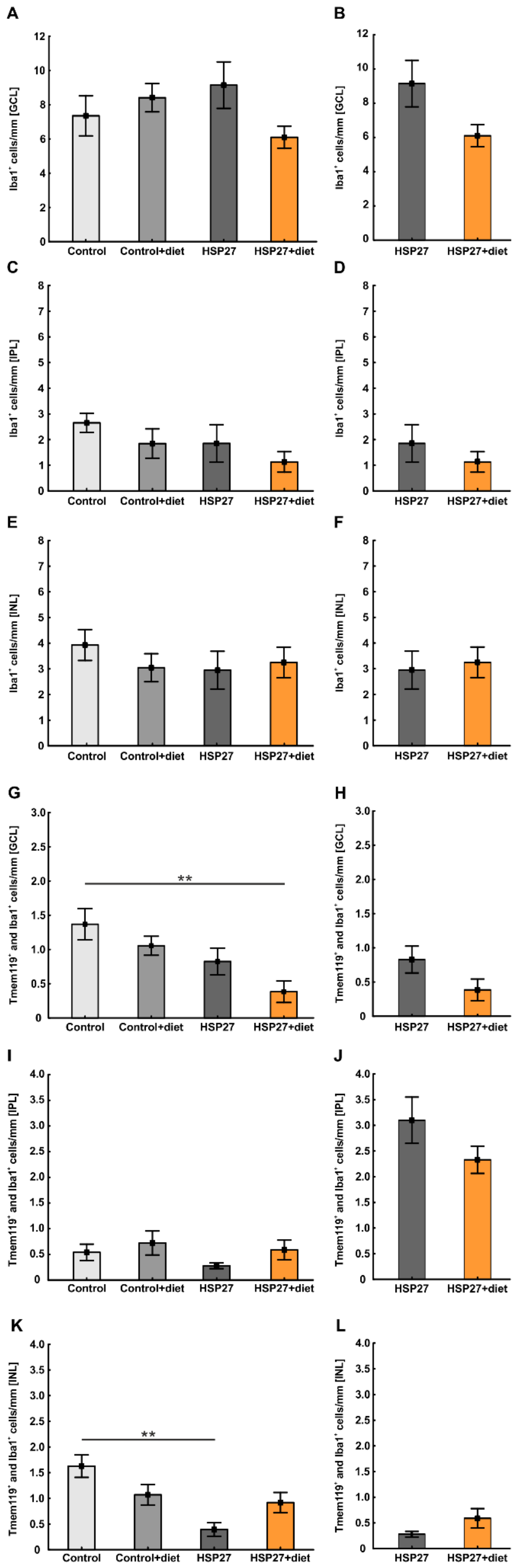

A
B
C
D
E
F
G
H
I
J
K
L
Iba1+ cells/mm [GCL]
Iba1+ cells/mm [IPL]
Iba1+ cells/mm [INL]
Tmem119+ and Iba1+ cells/mm [GCL]
Tmem119+ and Iba1+ cells/mm [IPL]
Tmem119+ and Iba1+ cells/mm [INL]
Control
Control+diet
HSP27
HSP27+diet
**

Supplementary Figure 2. The number of Iba1$^+$ microglia/macrophages was not altered within the groups in the GCL **(A+B)**, IPL **(C+D)**, and in the INL **(E+F)**. **(G)** The number of Tmem119$^+$ and Iba1$^+$ microglia in the GCL was significantly lower in HSP27+diet retinas compared to controls ($p<0.010$). **(H)** No difference was noted in the number of Tmem119$^+$ and Iba1$^+$ cells in the GCL between HSP27+diet and HSP27 samples. **(I+J)** In the IPL, similar numbers were observed regarding the number of Tmem119$^+$ and Iba1$^+$ cells. **(K)** In the INL, Tmem119$^+$ and Iba1$^+$ cell numbers were significantly lower in HSP27 retinas compared to control ones ($p<0.010$). **(L)** The number of Tmem119$^+$ and Iba1$^+$ cells was similar between HSP27+diet and HSP27 eyes in the INL. n=6 eyes/group. Values are mean±SEM. **$p<0.010$.

Supplementary Table 1: Proteins detected in the serum of no diet and diet mice. Values are mean±SEM. No diet values were set to 100%. In addition, mean raw intensity (RI) data are displayed. Significant p-values are marked in bold.

Protein	No diet [%]	No diet (RI)	Diet [%]	Diet (RI)	P-value
CD30	100.00±30.85	14951.3333	239.05±99.88	18160.4609	0.066
CXCL13	100.00±14.71	11875.9167	163.89±12.22	14396.2891	**0.020**
Eotaxin-1	100.00±20.24	12588.4167	134.99±43.08	14689.3125	0.128
Eotaxin-2	100.00±28.22	13009.8333	99.70±28.68	14672.2266	0.810
Fas ligand	100.00±99.92	11419.5833	-150.13±187.08	12106.4688	0.575
Fraktaline	100.00±112.64	11185.1667	-376.83±316.51	11602.7344	0.230
GCSF	100.00±29.24	15713.75	44.86±12.94	14893.8594	0.230
GM-CSF	100.00±47.25	11863.5	-474.67±228.28	9338.98828	**0.031**
IFN-γ	100.00±105.43	11994.25	-463.20±221.63	9217.87891	**0.045**
IL-1α	100.00±2500.83	11250.0833	-22004.20±11285.86	9505.4375	0.066
IL-2	100.00±117.82	11505	892.15±403.70	8624.03906	**0.045**
IL-3	100.00±1975.52	10805.4167	-9631.09±4364.25	8763.57422	**0.045**
IL-9	100.00±130.54	11339.5833	-1118.84±509.15	8723.35156	**0.045**
IL-10	100.00±142.46	14706	1339.08±574.02	11539.9805	0.065
IL-12 p40/70	100.00±110.94	11850.5	-2369.61±993.22	8725.33984	0.066
IL-12 p70	100.00±18.50	11719.25	-40.52±36.52	9047.27344	**0.005**
IL-13	100.00±148.20	10678.3333	-863.73±522.49	8633.66406	**0.045**
IL-17a	100.00±487.78	11233.5833	-1622.09±910.84	8584.17969	0.066
I-TAC	100.00±98.25	15526.4167	313.08±275.70	11516.3164	0.936
KC	100.00±47.89	11725.9167	-35.86±136.60	10179.4258	0.689
Leptin	100.00±144.23	11533.3333	-21.05±349.59	9821.60938	0.230
LIX	100.00±20.50	10606.9167	62.56±19.85	11181.9688	0.230
Lymphotactin	100.00±57.26	12808.3333	-88.24±142.38	12859.8398	0.378
MCP-1	100.00±40.95	10864.6667	-80.71±93.79	13499.0664	0.093
MIG	100.00±192.75	18361.5	-738.20±681.41	17154.4961	0.173
MIP-1γ	100.00±15.12	12723.6667	47.97±5.20	12233.2969	**0.008**
RANTES	100.00±1523.56	13380.0833	-1664±1570.68	11815.6445	0.173
SDF-1α	100.00±96.93	14915	39.89±66.73	12877.5273	0.093
I-309	100.00±92.39	11519.0833	152.73±57.58	11365.9609	0.689
TECK	100.00±205.34	11521.8333	-198.03±215.51	10054.5625	0.093
TIMP-1	100.00±156.14	30153.8333	-196.51±168.64	20916.1758	0.066
TIMP-2	100.00±742.57	10555.2348	1052.16±657.79	11439.5781	0.093
TNF-α	100.00±54.00	12755.8325	111.36±20.36	14061.8555	0.810
TNFR1	100.00±30.42	12841.7259	106.70±15.71	16709.1367	0.936
TNFR2	100.00±33.85	9690.97417	88.70±11.21	14804.1172	0.810

Supplementary Table 2: List of proteins assigned to KEGG pathways identified in the analysis. Each row indicates the pathway name(s) and the associated proteins detected in the dataset.

Pathways	Proteins per pathway	Protein
JAK-STAT signaling pathway/Cytokine-cytokine receptor interaction	7	GM-CSF
		IFN-γ
		IL-2
		IL-3
		IL-9
		IL-12
		IL13
Pathways in cancer	5	IFN-γ
		IL-2
		IL-3
		IL-12
		IL13
Th1 and Th2 cell differentiation/Inflammatory bowel disease	4	IFN-γ
		IL-2
		IL-12
		IL-13
Fcε RI signaling pathway	3	GM-CSF
		IL-3
		IL-13
T-cell receptor signaling pathway	3	GM-CSF
		IFN-γ
		IL-2
Coronavirus disease	3	GM-CSF
		IL-2
		IL-12
Amoebiasis	3	GM-CSF
		IFN-γ
		IL-12
Asthma	3	IL-3
		IL-9
		IL-13
Allograft rejection/Type I diabetes mellitus/Chagas disease	3	GM-CSF
		IL-2
		IL-12
IL-17 signaling pathway	3	GM-CSF
		IFN-γ
		IL-13
Measles/C-type lectin receptor signaling pathway	2	IL-2
		IL-12

Graft-versus-host disease/Th17 cell differentiation	2	IFN-γ
		IL-2
Tuberculosis/Herpes simplex virus 1 infection/Malaria/Leishmaniasis/Influenza A/African trypanosomiasis/Toxoplasmosis	2	IFN-γ
		IL-12
Natural killer cell mediated cytotoxicity/Rheumatoid arthritis	2	GM-CSF
		IFN-γ
Acute myeloid leukemia/Hematopoietic cell lineage/Human T-cell leukemia virus 1 infection	2	GM-CSF
		IL-2
PI3K-Akt signaling pathway	2	IL-2
		IL-12
Transcriptional misregulation in cancer	2	GM-CSF
		IL-3
TNF signaling pathway/Kaposi sarcoma-associated herpesvirus infection/Shigellosis	1	GM-CSF
Fluid shear stress and atherosclerosis/Systemic lupus erythematosus/HIF-1 signaling pathway/TGF-beta signaling pathway/Osteoclast differentiation/Proteasome/PD-L1 expression and PD-1 checkpoint pathway in cancer/Necroptosis/Hepatitis C/Antigen processing and presentation	1	IFN-γ
Autoimmune thyroid disease/Intestinal immune network for IgA production/Viral protein interaction with cytokine and cytokine receptor/Yersinia infection	1	IL-2
Apoptosis	1	IL-3
Legionellosis/Alcoholic liver disease/Toll-like receptor signaling pathway/Lipid and atherosclerosis/Pertussis/RIG-I-like receptor signaling pathway	1	IL-12
